# The effect of a telerehabilitation virtual reality intervention on functional upper limb activities in people with multiple sclerosis: a study protocol for the TEAMS pilot randomized controlled trial

**DOI:** 10.1186/s13063-020-04650-2

**Published:** 2020-08-12

**Authors:** Alon Kalron, Anat Achiron, Massimiliano Pau, Eleonora Cocco

**Affiliations:** 1 Department of Physical Therapy, School of Health Professions, Sackler Faculty of Medicine, Tel-Aviv University, Tel-Aviv, Israel; 2 Sagol School of Neuroscience, Tel-Aviv University, Tel-Aviv, Israel; 3 Multiple Sclerosis Center, Sheba Medical Center, Tel Hashomer, Israel; 4 Sackler Faculty of Medicine, Tel-Aviv University, Tel-Aviv, Israel; 5 Department of Mechanical, Chemical and Materials Engineering, University of Cagliari, Cagliari, Italy; 6 Multiple Sclerosis Center, Binaghi Hospital, Department of Medical Science and Public Health, University of Cagliari, Cagliari, Italy

**Keywords:** Multiple sclerosis, Telerehabilitation, Upper limb, Virtual reality, Pilot randomized controlled trial

## Abstract

**Background:**

Approximately 60% of people with multiple sclerosis (PwMS) suffer from upper limb dysfunction. Our primary goal is to implement a single-blind, randomized control trial (RCT) designed to compare the effectiveness of an 8-week home-based telerehab virtual reality (VR) program with conventional therapy in PwMS with manual dexterity difficulties. Secondary aims include (a) evaluating the impact of the programs on quality of life after the intervention and a follow-up 1 month later and (b) evaluating the impact of the programs on adherence and satisfaction.

**Methods:**

Twenty-four PwMS will be recruited to the study which will be conducted at two established MS centers: (1) The Regional Center for Diagnosis and Treatment of Multiple Sclerosis, Binaghi Hospital, Cagliari, Italy, and (2) The Multiple Sclerosis Center, Sheba Medical Center, Tel-Hashomer, Israel. Participants will complete a total of three assessments focusing on upper limb functions. Both groups will receive 16 training sessions focusing on functional upper limb activities. The home-based telerehab VR intervention will comprise a custom-made software program running on a private computer or laptop. PwMS will perform several activities of daily living (ADL) functions associated with self-care, dressing, and meal preparation. Conventional therapy will focus on task-related upper-limb treatments while in a sitting position, indicative of the standard care in MS. Following 8 weeks of training, participants will complete a further outcome assessment. The same tests will be conducted 1 month (as a follow-up) after completion of the intervention.

**Discussion:**

The outcomes of this study have tremendous potential to improve the quality of evidence and informed decisions of functional upper limb activities in PwMS. If comparable results are found between the treatments in improving upper limb outcomes, this would suggest that PwMS can choose the program that best meets their personal needs, e.g., financial concerns, transportation, or accessibility issues. Secondly, this information can be used by healthcare providers and medical professionals in developing upper limb exercise programs that will most likely succeed in PwMS.

**Trial registration:**

ClinicalTrials.gov NCT04032431. Registered on 19 July 2019.

## Background

Multiple sclerosis (MS) is a chronic, progressive disease of the central nervous system afflicting over 2.5 million people worldwide [1]. One of the most common reported complications is upper limb dysfunction. Several studies have described a high percentage of upper limb dysfunction in people with MS (PwMS), ranging from 50 to 76% [2, 3], which can start very early after disease onset. Upper limb dysfunction in PwMS reduces the ability to perform activities of daily living (ADLs) [4], resulting in decreased independence [5].

At some crucial point, upper limb dysfunction leads to irreversible disability resulting in secondary health conditions that are difficult, if not impossible, to treat [6]. This progression can result in decreased social, recreational, and vocational participation and ultimately to a poorer quality of life [7, 8]. The decrease in participation and work productivity combined with an increase in secondary health conditions has significantly impacted global costs [9, 10]. In Italy, at present, the rehabilitation expenditures of the MS population are approximately 27% of the direct healthcare costs of their National Health System [11].

Exercise training can improve functional activities of the upper limb and perhaps decrease the rate and extent of disability in PwMS [12–18]. Facility-based upper limb training programs, whether in a healthcare setting or laboratory, have yielded beneficial outcomes for PwMS [13–16]. However, lack of access to these programs, especially if one lives in a remote area where there are fewer options or where there are healthcare/medical facilities but no MS experts, may render it difficult to engage in traditional healthcare facility upper limb exercise training programs.

Telerehabilitation (telerehab) has the potential for providing upper limb training for PwMS in the home environment. However, its success depends on ongoing communication with knowledgeable and experienced exercise personnel who assist in the safe administration of these exercises. Telerehab strategies include videoconferencing (e.g., via Skype™), remote monitoring of signs and activity (e.g., via electronic monitors), and distribution of specialized and individualized information via electronic mechanisms (e.g., email or Internet posting of newsletters or videos). Telerehab can employ any or all of these strategies with the aim of bridging the gap between the diverse needs reported by PwMS and specialized MS care. Telerehab has proven beneficial for PwMS by increasing their physical activities (i.e., daily walking), decreasing fatigue, improving cognitive function, mobility, balance, participation, and quality of life [19]. Therefore, it is surprising that telerehab, known to improve functional activities of the upper limbs in MS, has been essentially ignored.

Importantly, the number of older adults with MS is at an all-time high and continually increasing [20]. This population suffers from upper limb dysfunction, poor health status, depression, loneliness, cognitive difficulty, and a dependency on others to perform their ADLs [21]. As a result, their prospects of leaving the home environment to receive treatment elsewhere are rapidly diminishing. Although the cost of rehab homecare is very expensive, utilizing telerehab for this specific segment of society should be a top priority.

A promising relatively new component of telerehab is virtual reality (VR). VR offers the opportunity to receive high-intensity, task-oriented, multisensorial feedback training. Trials investigating the benefits of VR in PwMS have shown promising results in walking improvement and balance [22]. VR has been suggested as a more motivational and cost-effective alternative to standard care and has been proven to improve upper limb function [23, 24] mainly in stroke survivors undergoing neurorehabilitation. Unfortunately, the use of VR for upper limb training is rare in PwMS, which is quite discouraging, since findings from the few studies examining this rehabilitation option have demonstrated considerable improvement in upper limb movements following training [25, 26].

Therefore, the primary goal of the TEAMS (TElerehabilitation And Multiple Sclerosis) is to implement a single-blind, RCT designed to compare the effectiveness of an 8-week home-based telerehab VR program with conventional therapy in PwMS with manual dexterity difficulties. Secondary aims include (a) evaluating the impact of the programs on quality of life after the intervention and follow-up 1 month later and (b) evaluating the impact of the programs on adherence and satisfaction. A tertiary aim will be to generate effect sizes which can be used to power a larger clinical trial aimed at improving upper limb functional activities via telerehab in PwMS in Italy and Israel. We hypothesize that there will be no difference in outcomes between a home-based telerehab VR training program aimed at improving functional activities of the upper limbs compared with a conventional clinic-based program after the intervention and follow-up 1 month later. We also intend to corroborate a secondary hypothesis that performing the telerehab VR training will lead to better outcomes in quality of life and adherence to upper limb exercise.

## Methods/design

### Study design and settings

**Fig. 1:**
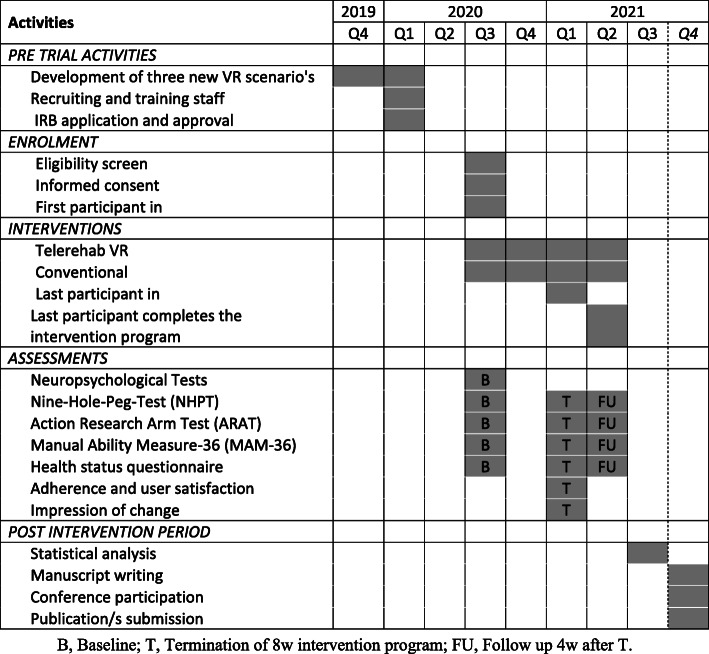
Study schedule

The study is a prospective, assessor blinded, parallel group pilot RCT. Figure 1 illustrates the study schedule. The implemented study design will adhere to the CONSORT guidelines [27] and will be conducted at two established MS centers:

1. The Regional Center for Diagnosis and Treatment of Multiple Sclerosis, Binaghi Hospital, Cagliari, Italy
2. The Multiple Sclerosis Center, Sheba Medical Center, Tel-Hashomer, Israel

### Methodology and work plan

Approval will be obtained from each Institutional Review Board Committee (IRB) prior to commencement. All participants will sign an informed consent form and will be randomly assigned to one of the two intervention groups:

1. Telerehab VR training
2. Conventional therapy

A block randomization procedure will be performed by the study coordinator who will place numbered tickets in sealed opaque envelopes. The sealed envelopes will be opened sequentially by the investigator only after the participant’s name and other details will be written on the appropriate envelope. The participants will be informed as to the resulting group allocation before the pre-intervention tests. The assessor will then assign the participants to an intervention program. The two interventions will be comparable in length (8 weeks), frequency (twice weekly), and session duration (50–60 min). The 8-week intervention period is in accordance with other studies investigating upper limb physical rehabilitation in PwMS [2].

**Fig. 2:**
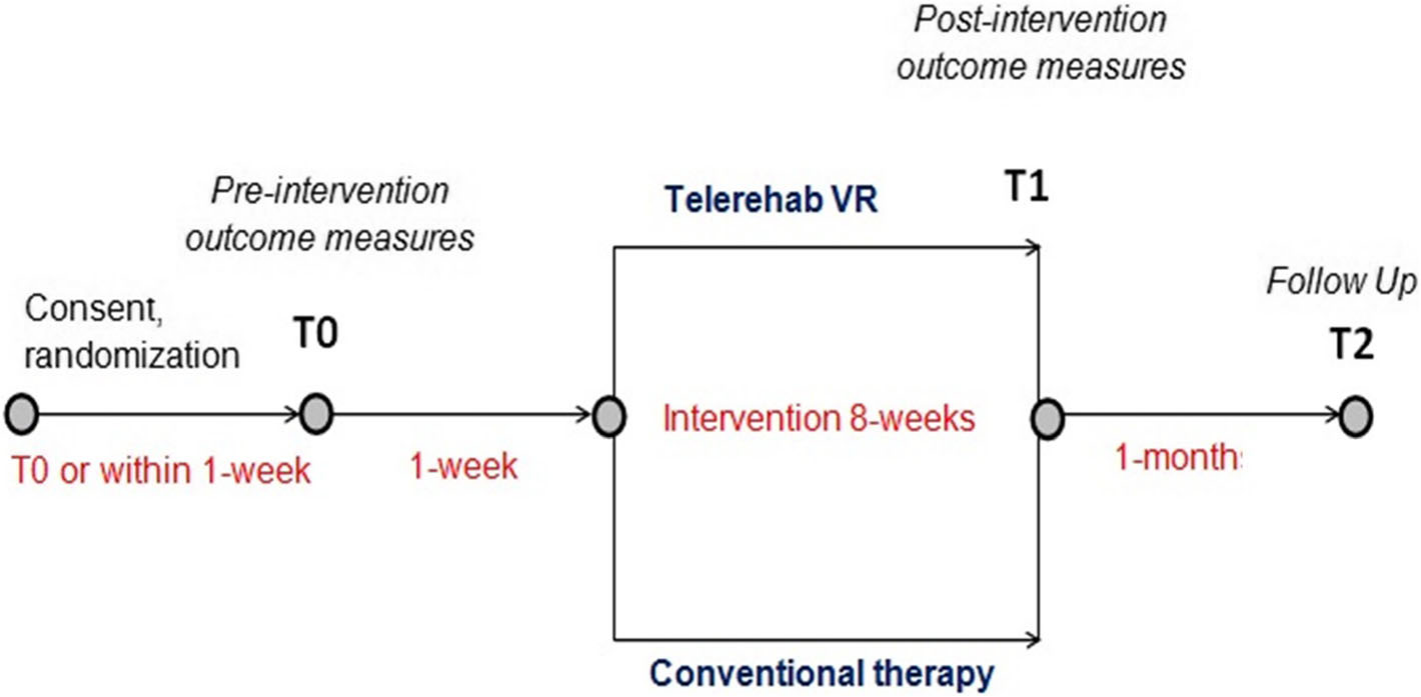
The procedures that participants will undergo at T0, T1, and T2

Pre-intervention tests (T0) characterizing groups and obtaining baseline values of primary and secondary outcome measures will be performed 1 week ± 3 days prior to the intervention program. Within 1 week ± 3 days after completion of the intervention, post-intervention tests will be performed (T1). The same tests will be conducted at a 1 month±3 days follow-up, (T2) after completion of the intervention. During the follow-up period (T1-T2), patients will be instructed to continue their regular activities. All assessments and interventions will be performed at the two MS centers. Due to the nature of the intervention, therapists and participants will not be blinded to the group allocation; however, the assessor will be blinded. Figure 2 illustrates the procedures that participants will undergo at T0, T1, and T2.

### Participants

In total, 24 individuals with MS will be recruited from the two participating MS centers (12 from each center). Recruitment of participants will be handled by advertisement and awareness of the trial through the medical staff of each participating center.

#### Inclusion criteria

1. Diagnosis of MS according to the revised McDonald Criteria 2017 [28]
2. Aged 25–60 years old
3. An Expanded Disability Status Scale score ≥ 6 [29]
4. An ability to understand and execute simple instructions
5. A cut-off score of > 0.5 pegs/s (=18 s) on the nine-hole peg test (NHPT) (selected due to its high discriminative and predictive ability in distinguishing ADL independence in PwMS) [7]

#### Exclusion criteria

1. Orthopedic and other neurological disorders affecting upper limb movements (e.g., epileptic seizures)
2. Contra-indication to physical activity (e.g., heart failure, severe osteoporosis
3. Moderate or severe cognitive impairments as indicated by the Mini-Mental State Examination [30] score < 21
4. Pregnancy (self-reported)
5. Severe uncorrected visual deficits
6. MS clinical relapse or treatment with corticosteroid therapy within 90 days prior to enrollment
7. Started or stopped a disease-modifying therapy for MS within 90 days prior to enrollment
8. Patients who received a course of physical or occupational therapy (home, outpatient, or inpatient) within the past 30 days
9. Other treatments that could influence the effects of the interventions. PwMS eligible for participation will be informed of the study by their rehabilitation specialist, both orally and in writing. All participants will provide written informed consent.

#### Sample size

The sample size is based on Julious’s [31] recommendation that the rule of thumb for a pilot study is a sample size of 12 subjects per group. The justifications for this sample size are based on rationale relating to feasibility, mean and variance precision, regulatory considerations, and the expected change in the study’s primary outcome measure (NHPT). According to the literature, a 20% change in the NHPT demonstrates a clinically meaningful worsening in PwMS [32]. In our study, power will be set at 80% and alpha at 5%. Therefore, 24 subjects (12 in each group) will be required in order to detect differences between the two treatment groups (assuming non-inferiority with moderate correlations among covariates, *r*-squared = 0.50).

#### Interventions

Both interventions will focus specifically on functional upper limb exercise training and be administered by experienced physiotherapists or occupational therapists with at least 2 years of professional experience in the field of neurorehabilitation. Therapists at each site will receive training protocols to ensure homogeneity of treatment between sites.

#### Telerehab VR intervention

The telerehab VR intervention consists of a custom-made software program, that will run on a personal computer (or tablet), developed under Unity (Unity Technologies Inc., San Francisco, USA). Unity is a cross-platform game engine worldwide adopted to create three-dimensional, two-dimensional, virtual reality, and augmented reality games, as well as simulations and other experiences. PwMS will be requested to perform several ADLs from the three main areas of self-care, dressing, and meal preparation. Taking place in a realistic home scenario, the PwMS will pick up objects and move them to a predefined target clearly indicated on the screen. Hand and object trajectory are controlled by a low-cost non-contact infrared controller (Leap Motion). This instrument was proven successful in upper limb rehabilitation of individuals with neurologic and orthopedic conditions [33–35].

**Fig. 3:**
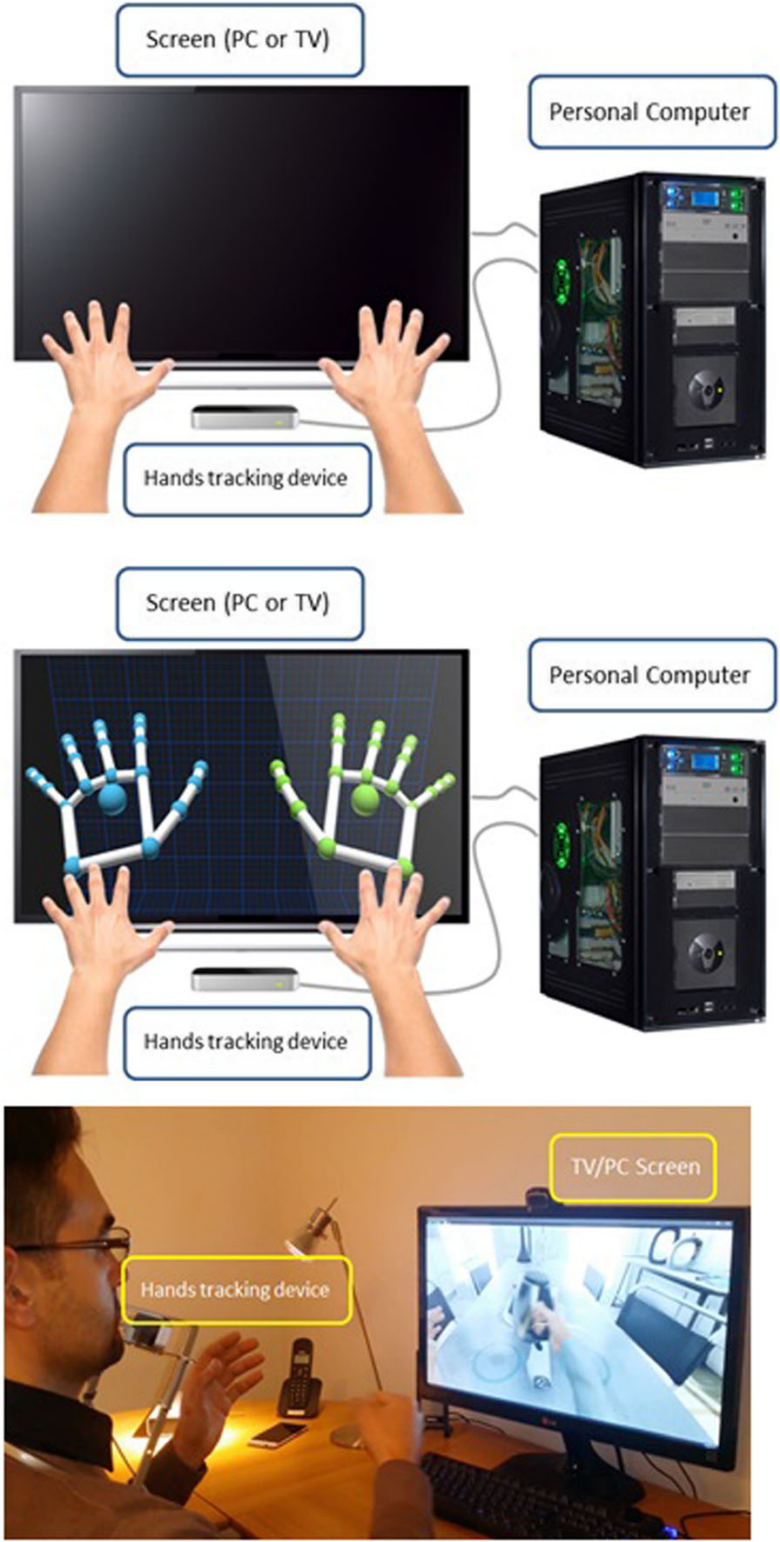
Schematic representation of the system

During the exercise, the hand coordinates will be continuously recorded; thus, data kinematic data calculated on the basis of the 3D trajectory like speed, accuracy on target placement, and movement smoothness will be accessible. This data will be stored in the PC/tablet and will also be sent remotely to the clinical center for further analysis/processing. Both target position and task complexity will define the exercise difficulty, which can be modified (i.e., increased or decreased) automatically on the basis of the previous performance or manually modified by the user. A suitable period of familiarization with the installation/management of the hardware and software will be carried out under the supervision of engineers and therapists to ensure that the exercises are being correctly performed. A schematic representation of the system is shown in Fig. 3.

#### Conventional therapy

Conventional therapy will focus on task-related upper-limb treatments while in sitting denoting the standard care for PwMS [2]. Several manual techniques, therapy tools and objects of ADL will be allowed during treatment. Use of additional electrical or mechanical therapy devices (i.e., support arm systems, splints) will be avoided. The interventions will be conducted on a one-on-one basis in the physiotherapy or occupational therapy department of each participating center. Training and therapy content will be tailored to each participant’s preferences, the agreed movement aims and their motor function level.

#### Safety considerations and adverse events

Adverse events occurring during and between treatments will be recorded and compared between groups (without statistical comparisons). Training and all measurements are non-invasive and place the subject at no risk other than those that normally may occur during functional upper limb activities. Nevertheless, the patient will be free to withdraw from the study at any time. The investigator has the right to withdraw a patient from the study due to any reason concerning the health or well-being of the patient, or in case of lack of cooperation. The reason for any withdrawal will be noted in the patient’s hospital file.

### Outcomes

#### Primary outcomes (collected at T0, T1, T2)

##### NHPT

The NHPT was selected based on the widespread adoption and extensive data available. Furthermore, the NHPT is recommended as a gold standard for measuring manual dexterity in PwMS [32]. The NHPT has excellent psychometric properties as to reliability, discriminant, concurrent, and ecological validity; can detect progression over time; is sensitive to treatment; and, as such, is recommended for inclusion in clinical trials. Briefly explained, the NHPT requires participants to repeatedly place nine pegs into nine holes, one at a time, as quickly as possible and then remove them from the holes. The total time needed to complete the task is then recorded. Two consecutive trials with the dominant hand are immediately followed by two consecutive trials with the non-dominant hand [4, 8].

#### Secondary outcomes (collected at T0, T1, T2)

##### Action Research Arm Test (ARAT)

The ARAT is a 19-item observational measure used by physical therapists and healthcare professionals to assess upper extremity performance in terms of coordination, dexterity, and functioning in several neurologic conditions, including MS [36]. Items comprising the ARAT are categorized into four subscales (grasp, grip, pinch, and gross movement) and arranged in order of decreasing difficulty, with the most difficult task examined first, followed by the least difficult task. Task performance is rated on a 4-point scale, ranging from 0 (no movement) to 3 (movement performed normally).

##### Manual Ability Measure-36 (MAM-36)

The MAM-36 is a questionnaire based on perceived ease or difficulty that a person may experience when performing unilateral and bilateral ADL tasks. During a semi-structured interview, the subject is asked to rate 36 unilateral and bilateral ADL tasks using a 4-point scale. The scores of the different tasks are summed up and transformed using a Rasch-derived conversion table. The MAM-36 has adequate psychometric properties and is recommended as an outcome measure for upper limb function in PwMS [37].

##### Health status questionnaire (SF-36)

The Short Form-36 is one of the most widely used generic measures of health-related quality of life and has been shown to discriminate between subjects with different chronic conditions and between subjects with different severity levels of the same disease. The instrument addresses health concepts relevant to MS patients from the patient’s perspective. Availability of normative data makes the SF-36 beneficial for comparative purposes. There is considerable evidence of the validity of the SF-36 in PwMS [38].

##### Adherence and user satisfaction

Adherence to exercise will be evaluated at the end of the intervention phase by examining the patient’s exercise diaries. The User Satisfaction Evaluation Questionnaire (USEQ) will be used to assess the participants’ satisfaction with the training program [39]. The USEQ is a reliable questionnaire with internal consistency designed to evaluate the satisfaction of the user in virtual rehabilitation systems.

##### Impression of change (collected only at post-intervention T1)

A 7-point Likert-type global rating scale from both the patient and therapist’s perspective will be applied. The question that will be asked will be “Compared to before treatment, at present, how would you rate your/the participant’s functional upper limb activities?” The responses will be rated as 1 = worse than ever, 2 = much worse, 3 = slightly worse, 4 = unchanged, 5 = slightly improved, 6 = much improved, and 7 = greatly improved.

##### Brief Repeatable Battery of Neuropsychological Tests (BRB-N) (collected only at baseline T0)

The BRB-N are sensitive measurement tools aiding in identifying cognitive impairment in PwMS. This battery of tests includes the Selective Reminding Test, the 10/36 Spatial Recall Test, the Symbol Digit Modalities Test, the Paced Auditory Serial Addition Test, and the Word List Generation Test [40]. Cognitive status will be collected only at baseline*.*

### Statistical analysis

Data analysis will be performed by the SPSS 25.0 program. The data will be initially examined for normality violations, outliers, errors, and patterns of missing values; missing data will be replaced by multiple imputation techniques found in the SPSS. In the event of exacerbations, the analysis will be conducted with and without the individuals who had relapsed in order to identify any untoward effects on the outcomes. The data analysis itself will follow intent-to-treat principles. The effect of the intervention on the NHPT (aim 1), ARAT (aim 1), MAM-36 (aim 1), SF-36 (aim 2), and adherence and user satisfaction (aim 3) will be examined using the Condition × Time mixed model multivariate analysis of variance (MANOVA) followed by an inspection of the univariate F-ratios. Conditions will be a between-subjects factor and time and a within-subjects factor. Interactions and main effects will be further calculated using post hoc analyses with a correction of alpha. Effect sizes associated with univariate *F*-statistics will be expressed as eta-squared (*η*^2^), and effect sizes based on a difference in mean scores will be expressed as Cohen’s *d*. We will include cognitive scores (BRB-N) as covariates in all final statistical analyses.

### Roles and mode of integration between the teams

Data will be shared throughout the project by personal meetings, Skype™ video calls, conference calls, and e-mail correspondence. Specifically, the Italian group will be responsible for designing and implementing three new VR scenarios relating to functional activities of the upper limb. The new VR scenarios will be planned in conjunction with the Israel group who will also play an active role in the quality assessment procedures of the developed elements. Both groups will participate equally in the pilot RCT. Roles include recruitment and guidance of patients and therapists, implementing the telerehab VR system in the patient’s home with multi-layer security, end-to-end encryption, assessment of outcome measures, and data collection. Both sites will contribute to the analysis and interpretation of data and dissemination of the findings. An additional role of the Israel team will be to prepare a user manual detailing how to use the system and providing clinical guidelines/recommendations for both the therapist and patient. Both groups will be responsible for data entry, coding, security and storage. No anticipated harm is expected; therefore, a data monitoring committee is not required. However, an independent biostatistician will be involved in the data analysis. Periodic reports on progress of the trial will be provided to the funding bodies. There is no intended use of professional writers.

## Discussion

The TEAMS project has tremendous potential to improve the quality of evidence and informed decisions of functional upper limb activities in PwMS. The project addresses the needs of PwMS [2, 6, 8], specifically, if they are provided access to functional upper limb exercises and does the location where the exercises are performed impact their outcomes? Is it necessary to travel to a healthcare facility with access to equipment and MS experts in order to benefit from upper limb exercises or can the PwMS safely and effectively exercise in their own home, if provided with expert guidance? We will compare a conventional healthcare facility training program with a VR telerehab home-based program. This information will impact the PwMS’s informed decisions as to how to spend their time and potentially limited resources.

If comparable results are found between the treatments in improving upper limb outcomes, this would suggest that PwMS can choose the program that best meets their personal needs, e.g., financial concerns, transportation, or accessibility issues. Secondly, this information can be used by healthcare providers and medical professionals by developing upper limb exercise programs that will most likely succeed and can then be distributed to neurologists and other medical providers. Finally, increasing an individuals’ accessibility to upper limb exercise options may prevent or slow the decline of disability, increase independence in ADL functions, and help maintain a better quality of life.

The telerehab VR proposal for restoring upper limb functional activities in PwMS is an expansion of a project originally developed by the University of Cagliari (Italy) that included a feasibility study which produced encouraging results. The following goals were reached during the initial phase: (1) recruitment of technological developers and MS professionals, (2) definition of upper limb functional difficulties and relevant rehabilitation exercises of the population target, and (3) development of a hardware-software VR platform based on low-cost tools, encouraging self-practice of functional exercises of the upper limb. Important elements include a user-friendly system that can be easily assembled, used in the home and be monitored from a distance by a medical/rehab professional.

To date, the system includes a single VR scenario (kitchen scene) simulating the use of a kettle in the kitchen. Feasibility and safety of the VR platform were explored by five PwMS who were asked to perform a 30-min session under the supervision of neurologists, therapists, and bioengineers in both the home and laboratory setting. Our preliminary data revealed that the system can be installed and easily operated on a variety of electronic devices (private computer, tablet, smart TV) and can be controlled from a distance. Notably, the cost of the platform is affordable and designed to employ “off-the-shelf” components (e.g., Leap Motion controller) and royalties-free software (Unity).

Despite these achievements, in order to reach full potential, this novel project requires further development and collaboration. In addition to the multicenter (Italy, Israel) pilot RCT, we intend to (1) improve the platform by developing new VR scenarios encompassing important functional activities of the upper limbs (e.g., self-care, dressing, meal preparation) and (2) implement the remote transmission of data relating to upper limb kinematics to the clinical center on the basis of the specific movements performed during the exercise sessions. Collaborating with experienced and knowledgeable personnel from Italy and Israel and researching PwMS from different countries and backgrounds, including their various infrastructures and health systems, are the main strengths of the project.

Nevertheless, several limitations of the project are worth noting. First, in the virtual scenario, there is no sensory feedback; therefore, some may argue that the transfer of learning actual activities from the virtual exercises is questionable. We acknowledge that sensory feedback (besides visual) in VR is a major challenge [41]. Although there are several emerging methods for adding the sense of touch to VR, the current costs prevent usage of these devices in home-based treatment. However, we are confident that with the rapid development of the field of VR and telemedicine, future phases of our project will include improved systems that provide sensory feedback. Second, the conventional therapy and telerehab VR therapy are not exactly matched in terms of exercises and activities. However, we believe that although there might be some diversity between the programs, we feel that it might strengthen the ecological validity of our findings. Furthermore, we believe that it reflects, in a more realistic manner, the current status of upper extremity rehabilitation of PwMS. Nevertheless, the two interventions will be comparable in length (8 weeks), frequency (twice weekly), and session duration (50–60 min). Finally, the telerehab VR system cannot guarantee that the patient themselves performed the training session or that another individual had replaced them. To date, this drawback is common with most (if not all) home-based training/game programs.

In conclusion, the proposed research complies with three highlighted goals: (1) encouraging scientific and technological co-operation between Italy and Israel, (2) improving patient access to care by receiving therapy beyond the physical walls of a traditional healthcare facility, and (3) tele-rehabilitation platform development and feasibility testing entailing technology innovation will lead to their integration into routine clinical treatment.

## Trial status

The current approved version of the protocol is version 2.0, December 12, 2019. The trial has yet not begun. The first patient enrollment is planned for July 2020. Recruitment will be completed by September 2021.

## Data Availability

The datasets used and/or analyzed during the current study will be available from the corresponding author upon reasonable request.

## Abbreviations

MS: Multiple sclerosis
ADL: Active daily living
PwMS: People with multiple sclerosis
VR: Virtual reality
RCT: Randomized controlled trial
NHPT: Nine Hole Peg Test
ARAT: Action Research Arm Test
MAM-36: Manual Ability Measure-36
SF-36: Health status questionnaire
BRB-N: Brief Repeatable Battery of Neuropsychological Tests
MANOVA: Multivariate analysis of variance
